# Rectourethral fistula after external beam radiotherapy for prostate cancer in a patient with thromboangiitis obliterans: A case report

**DOI:** 10.1097/MD.0000000000030343

**Published:** 2022-08-26

**Authors:** Keiichi Ohira, Kenta Konishi, Shuhei Aramaki, Ryo Kokubo, Kouhei Wakabayashi, Masanori Hirata, Michiko Imai, Katsumasa Nakamura

**Affiliations:** a Department of Radiology, Hamamatsu University School of Medicine, Shizuoka, Japan; b Department of Radiation Oncology, Iwata City Hospital, Shizuoka, Japan.

**Keywords:** adverse event, prostate cancer, radiotherapy, rectourethral fistula, thromboangiitis obliterans

## Abstract

Thromboangiitis obliterans (TAO) is a rare disease of unknown cause that causes segmental vasculitis in peripheral blood vessels. It is uncertain whether its presence causes serious adverse events in patients receiving external beam radiotherapy.

**Patient concerns:**

A 73-year-old Japanese man with prostate cancer underwent external beam radiotherapy.

**Diagnosis:**

After completion of radiotherapy, fingertip pain occurred, leading to the diagnosis of TAO.

**Interventions:**

The patient was instructed to stop smoking, but was unable to do so.

**Outcomes:**

Nine months after the completion of radiotherapy, fecaluria appeared, and a rectourethral fistula was diagnosed by contrast enema. The patient’s TAO was poorly controlled, and the patient died from aspiration pneumonia 33 months after completion of the radiotherapy regimen. No tumor recurrence was observed during this process, and there were no risk factors other than TAO that may have formed a rectourethral fistula.

**Lessons:**

This is the first report of rectourethral fistula caused by external beam radiotherapy for prostate cancer in which TAO was suspected to be involved. Although little is known about the relationship between TAO and radiotherapy, it should be noted that radiotherapy itself may increase the risk of normal tissue toxicity in patients with TAO.

## 1. Introduction

Thromboangiitis obliterans (TAO) is a rare disease of unknown cause that mainly causes segmental vasculitis in peripheral vessels. It is characterized by a predilection for the arteries of the lower extremities and is more common in men in their 20s to 40s, especially in smokers. The most common symptoms are coldness and paresthesia associated with peripheral ischemia in the extremities, but in rare cases, ischemia occurs in visceral vessels. Serious adverse events of radiotherapy have been reported in patients with vasculitis syndrome; however, few such events have been reported in individuals with TAO. We report a case of a patient with a rectourethral fistula after prostate cancer radiotherapy in which TAO was suspected to be involved.

## 2. Case report

**Figure 1.**
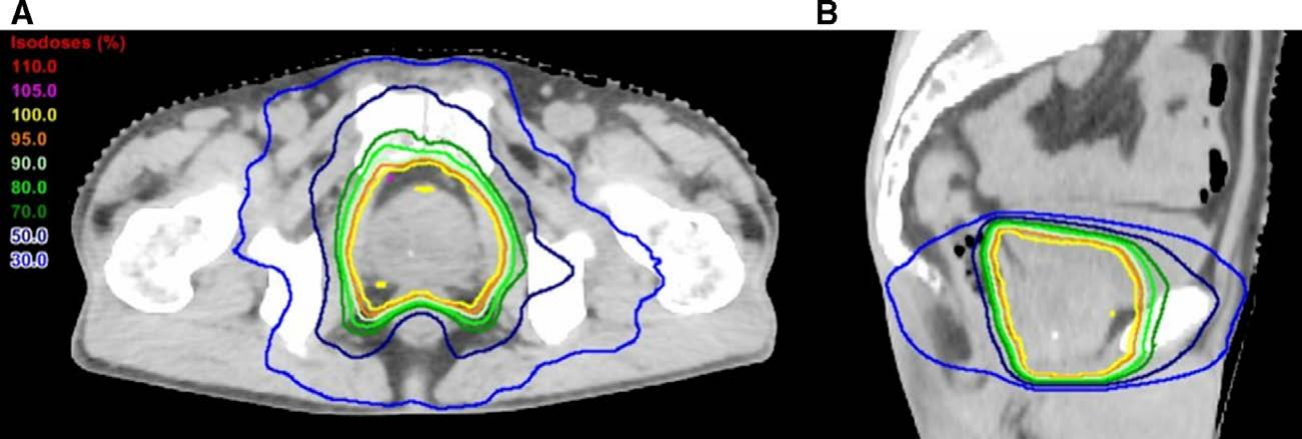
Dose distributions in the axial slice (a) and sagittal slice (b). The isodose lines indicate the percentage of the prescribed dose of 74 Gy.

A 73-year-old Japanese man was referred to our hospital because of a prostate-specific antigen (PSA) value of 837.8 ng/mL, and was diagnosed with prostate cancer (clinical stage T3aN0M0, Gleason score of 8). The patient’s medical history was unremarkable. After 4 months of hormone therapy, radiotherapy was performed with a total dose of 74 Gy in 37 fractions for the prostate and the proximal part of the seminal vesicles with volumetric modulated arc therapy (Fig. 1). The accumulated dose to the rectum and bladder were considered to be acceptable within the dose constraints widely recommended. No obvious adverse events were observed during radiotherapy.

Two months after the completion of radiotherapy, he reported experiencing pain in his fingertips and purpura at the fingertips. A vascular surgeon diagnosed TAO on the basis of the patient’s physical and computed tomography findings. The patient was instructed to stop smoking but was unable to do so.

Four months after the completion of radiotherapy, the patient complained of discomfort in the anus. Lower endoscopy revealed many ulcers on the anterior wall of the lower rectum. Steroid suppositories were prescribed on the basis of the diagnosis of radiation proctitis. However, the patient subsequently experienced pain in the toes, and antiplatelet agents and prostaglandin E1 were prescribed for this apparent exacerbation of TAO.

**Figure 2.**
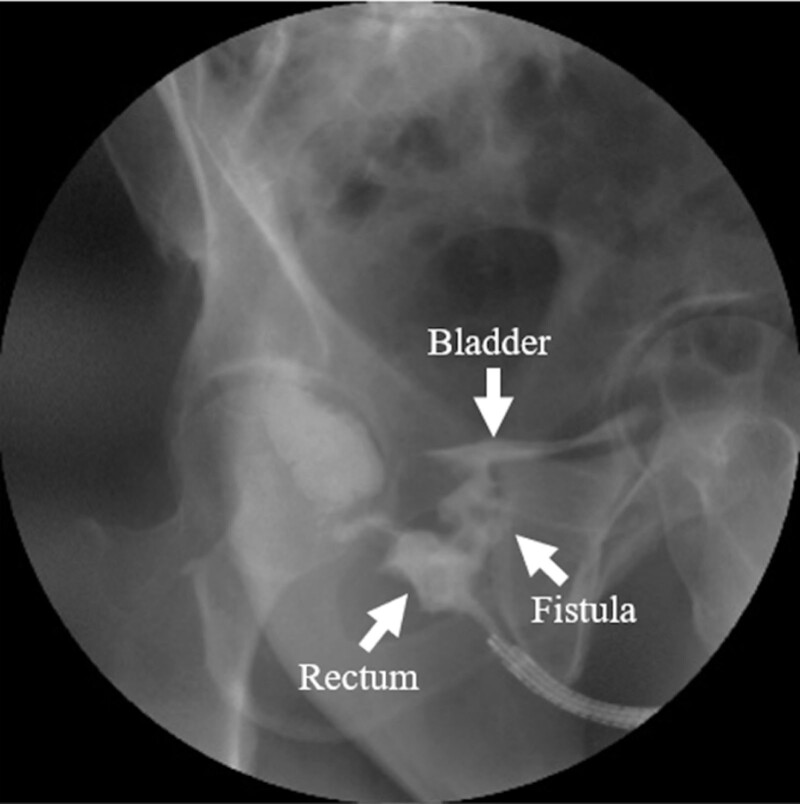
The fistula between the patient’s bladder and rectum, confirmed by contrast enema.

Nine months after the completion of radiotherapy, faecaluria developed, and after the administration of a contrast enema, a rectourethral fistula was diagnosed (Fig. 2). The fistula was treated with colostomy and cystostomy. The patient’s TAO was poorly controlled, and his clinical status declined because of ischemic necrosis of the toes. At 33 months after completion of the radiotherapy regimen, the patient died of aspiration pneumonia. During this process, there were no findings suggestive of tumor recurrence, including PSA level.

## 3. Discussion

Thromboangiitis obliterans, also known as Buerger disease, is a disease of unknown etiology that occurs mostly in young adult men. TAO has been described as rare in Western Europe and the United States but relatively common in Japan, Korea, Southeast Asia, and the Middle East.^[1]^ Although in some countries, the prevalence of TAO has apparently been decreasing in recent years. For example, the prevalence in Japan is now 7–10 cases per million, which is almost the same as that in Western countries.^[2]^ The initial symptoms of TAO are coldness, numbness, and pain in the extremities. As the disease progresses, ulcers and gangrene occur. It has been reported that >90% of TAO patients have a history of smoking. Smoking cessation is effective in controlling disease progression, and smoking often leads to progression.^[2–6]^ The effects of TAO are most common in the arteries of the extremities; however, lesions in visceral arteries such as mesenteric arteries, renal arteries, coronary arteries, and cerebral arteries have also been reported.^[7–11]^

There are no international criteria for TAO, but various diagnostic criteria have been proposed.^[1,12]^ In Japan, Shiono clinical criteria are often used: history of smoking, onset under the age of 50, occlusive lesions beyond the popliteal artery, involvement of the upper limb arteries or migratory phlebitis, and no risk factors for atherosclerosis other than smoking.^[13]^ It be easily diagnosed if a patient meets all the 5 criteria. However, in clinical practice, patients often meet only some of these criteria. Therefore, when Shiono criteria are used, TAO can be diagnosed if the patient’s clinical symptoms, vascular images, and histopathology are consistent, and differential diagnoses are ruled out.^[14]^

A rectourethral fistula occurred in the present patient. Rectourethral fistula is a quite rare adverse event of radiotherapy for prostate cancer, and has been reported to be caused by brachytherapy for prostate cancer. Shakespeare et al assessed 1455 patients treated with brachytherapy or brachytherapy combined with external beam radiotherapy to investigate the incidence of rectourethral fistulas, and they identified 3 (0.2%) patients with rectourethral fistulas,^[15]^ whereas Zelefsky et al evaluated rectal and urethral adverse events in 561 prostate cancer patients treated with 81 Gy intensity-modulated radiotherapy.^[16]^ Despite the relatively large number of patients, no grade 4 adverse events (including rectourethral fistula) were observed. To our knowledge, there have been no published reports on rectourethral fistulas induced by external beam radiotherapy alone for prostate cancer.

We speculate that the patient’s TAO itself enhanced the adverse events associated with radiotherapy. There is a case report by Barkhuysen et al that may support this hypothesis.^[17]^ They reported severe radiation osteonecrosis following radiotherapy for a squamous cell carcinoma of the soft palate in a patient with TAO. They applied an accelerated fractionation scheme with a total dose of 68 Gy given in 34 fractions, and grade 4 mucositis developed even at 28 Gy. They also reported that intractable intraoral ulcers developed 1 month after radiotherapy, and maxillary bone exposure and oral maxillary sinus fistulas developed 2 months after radiotherapy. Although the total dose was not markedly high, serious adverse events occurred from a relatively early period, as in our patient’s case. Barkhuysen et al hypothesized that normal tissues in TAO patients may have impaired the ability to recover from radiation damage, and they noted that autoantibodies such as anticollagen and antiendothelial cell autoantibodies are expressed in TAO patients as evidence of this.

There are scattered reports of adverse events associated with radiotherapy in patients with vasculitis, particularly those associated with collagen disease. Lin et al evaluated the adverse events of radiotherapy in 73 patients with collagen vascular diseases, and reported that all 73 patients had significantly more late adverse events and that the adverse events tended to be more severe compared to controls.^[18]^ Wo et al reviewed 3 case-controlled studies of 61, 38, and 36 cases and 1 retrospective study of 209 cases. They stated that there was no significant increased incidence of acute or late adverse events in the studies, but there was a trend toward more fatal late adverse events such as necrosis and obstruction of the bowel, bladder necrosis, and pericarditis.^[19–23]^ Although the precise risk and frequency of adverse events for collagen vascular diseases remain unclear, when clinicians consider using radiotherapy for patients with vasculitis, they should be aware that there can be a high risk of increased adverse events, and caution should be exercised in irradiating.

Regarding the potential mechanisms underlying such adverse effects, it has been speculated that microvascular damage caused by radiotherapy and collagen vascular disease might be additive.^[24]^ If so, it cannot be denied that TAO, which similarly produces thrombotic ischemia, may have the potential to cause serious side effects in radiotherapy. Of course, the mechanisms of vascular damage in TAO and other forms of vasculitis differ, and it may thus not be possible to directly compare the mechanisms. However, all these mechanisms have in common the fact that inflammation of blood vessels results in ischemia in the vessels’ dominant regions, and if the reduction of blood flow due to radiotherapy is combined with this, the recovery of normal tissues may be hindered.

The lack of reports on adverse events after radiotherapy in patients with TAO may be due to the rarity of this disease. Further investigations and accumulation of case reports are needed to establish the relationship between TAO and severe adverse events after radiotherapy.

## 4. Conclusions

We report the case of an elderly male smoker with TAO who developed a rectourethral fistula induced by radiotherapy. When patients with TAO are treated, oncologists should be aware of the potential increased risk of normal tissue toxicity and follow up carefully over the long term.

## Acknowledgments

The authors would like to thank KN International and PaperPal PreFlight service for English language editing.

## Authors’ contributions

Conceptualization: Keiichi Ohira, Katsumasa Nakamura

Investigation: Keiichi Ohira, Kouhei Wakabayashi, Michiko Imai

Supervision: Keiichi Ohira, Katsumasa Nakamura, Michiko Imai

Visualization: Keiichi Ohira

Writing—original draft: Keiichi Ohira

Writing—review & editing: Keiichi Ohira, Kenta Konishi, Shuhei Aramaki, Ryo Kokubo, Masanori Hirata, Katsumasa Nakamura.
